# Special Issue “Modern Analytical Strategies for Foodomics: From Nutritional Value to Food Security”

**DOI:** 10.3390/ijms27020611

**Published:** 2026-01-07

**Authors:** Cosima Damiana Calvano, Mariachiara Bianco

**Affiliations:** 1 Dipartimento di Chimica, Università degli Studi di Bari Aldo Moro, Via Orabona, 4-I-70126 Bari, Italy; mariachiara.bianco@uniba.it; 2 Centro Interdipartimentale SMART, Università degli Studi di Bari Aldo Moro, Via Orabona, 4-I-70126 Bari, Italy

Considering current research trends shaped by critical challenges in food science and the food industry, foodomics can be defined as a scientific discipline that investigates changes in the molecular composition and quality of foods arising from raw materials, processing, and cooking, as well as the effects of food consumption on human health and metabolism [1]. Food matrices are among the most challenging samples to examine because of their varied compositions and complex structural organization [2]. From physical, chemical, and biological perspectives, foods represent multifactorial systems composed of heterogeneous mixtures of macro- and micro-molecules often referred to as small molecules with molecular masses ≤1500 Da embedded within complex physical structures such as crystals, amorphous solids, aqueous solutions, gels, pores, cavities, organelles, and cells. Most foods consist primarily of four components: water and the three macronutrient classes including carbohydrates, proteins, and lipids alongside omnipresent plasticizers. Together, these components typically represent 70–99% of total food mass, despite the vast molecular diversity and heterogeneity within macronutrients [3]. Foodomics primarily focuses on these constituents, involving both qualitative and quantitative data analysis to recognize the specific effects of food on human health and nutrition [4,5,6]. To solve food complexity, advanced analytical chemistry [7,8,9,10], omics sciences [11,12,13,14], and bioinformatics [15,16,17] are jointly employed to address critical challenges in food quality, safety, and traceability [18,19,20], together with the complex relationship between diet and human health. In recent years, the constant evolution of analytical technologies has enabled a transition from traditional targeted approaches toward more comprehensive, sensitive, and high-throughput strategies.

This Special Issue, “Modern Analytical Strategies for Foodomics: From Nutritional Value to Food Security”, brings together five contributions that exemplify how state-of-the-art analytical tools can be effectively applied across different domains of food science, ranging from clinical nutrition to authenticity, allergen detection, dietary supplements, and emerging biosensing methodologies.

The clinical relevance of accurate food analysis is clearly demonstrated in the first study [21], on foods for special medical purposes (FSMPs); a microchip-based gel electrophoresis technique was developed for the rapid and reproducible determination of total nitrogen and protein profiles. The main issues in rapid, reproducible protein/nitrogen analysis involve matrix effects (such as non-protein nitrogen and protein aggregation in dairy), method limitations (Kjeldahl/Dumas time, hazardous reagents, differentiating N types), and the challenges in achieving both speed and accuracy, leading to the need for advanced techniques, for example, microfluidics or spectroscopies such as FT-NIR, to overcome traditional challenges in complex samples [22,23]. The work highlights substantial discrepancies between declared and experimentally measured nitrogen content, especially in liquid FSMPs, raising important concerns for patients with chronic kidney disease whose protein intake must be strictly controlled. This contribution underlines the pivotal role of robust analytical quality control in safeguarding vulnerable populations and ensuring the reliability of nutritional labeling.

The following two contributions [24,25] address food authenticity, employing a multi-analytical technique approach. By combining gas chromatography-based fatty acid profiling with rapid and non-destructive ^1^H NMR spectroscopy and multivariate data analysis, it was possible to significantly discriminate argan oils based on both region and preparation method [24]. The study represents a clear example of how complementary analytical platforms and chemometrics can be integrated to enable authenticity, purity control, and protection against food fraud. Dietary supplements represent another rapidly expanding sector that requires strict analytical surveillance [26]. In one contribution [25], ^1^H NMR spectroscopy and HPLC-DAD were used for quality control in supplements containing magnolia bark extract, a popular ingredient with documented anti-inflammatory and antioxidant properties. Although both techniques provided comparable quantitative results for magnolol and honokiol, significant discrepancies between declared and actual compositions were observed. Importantly, this study represents the first demonstration of ^1^H NMR as a routine quantitative tool for the control of magnolia-based supplements, highlighting the potential of spectroscopic techniques for fast, reliable, and cost-effective quality assessment.

Allergen contamination, one of the most critical aspects of food safety, is investigated in the third paper [27]. In the context of sustainable agriculture and green manuring practices, the potential cross-contamination of wheat with soybean and mustard allergens was investigated using a targeted bottom–up proteomics approach based on RPLC–MRM mass spectrometry [28,29]. The validated method enabled the reliable identification and quantification of specific marker peptides in complex processed matrices such as flour and cookies. This work demonstrates how modern targeted proteomics can effectively support regulatory compliance and consumer protection in the presence of emerging contamination scenarios linked to environmentally friendly farming practices.

Finally, the last contribution [30] provides a comprehensive review of immuno-polymerase chain reaction (IPCR) as an ultra-sensitive analytical platform for food contaminant detection. By merging the selectivity of immunoassays with the amplification capacity of PCR, IPCR has shown remarkable performance in the detection of a wide range of analytes, including pathogens, toxins, mycotoxins, allergens, pesticides, antibiotics, and persistent organic pollutants. The review critically discusses current formats, applications, and limitations while also outlining future perspectives on the implementation of IPCR in next-generation food safety monitoring.

Together, the papers collected in this Special Issue clearly demonstrate how modern analytical strategies, including chromatographic, spectroscopic, mass spectrometric, and bioanalytical methods, are reshaping the field of foodomics. These contributions not only highlight technological advancements but also emphasize their direct implications for nutrition, consumer safety, regulatory compliance, and food security. We believe that this Special Issue will provide valuable insights for researchers, analyst chemists, and stakeholders working at the interface of analytical science and food systems.
